# Left atrial volume, congestive heart failure, and obesity are associated with extent of left atrial fibrosis by late gadolinium enhancement

**DOI:** 10.1186/1532-429X-17-S1-P368

**Published:** 2015-02-03

**Authors:** Karl Grunseich, Bethlehem Mekonnen, Lauren A Simprini, Hamid Mojibian, Mark Marieb, Gourg Atteya, Daniel Cornfeld, Dana C Peters

**Affiliations:** 1 Yale University School of Medicine, New Haven, CT, USA

## Background

The cardiovascular MRI late gadolinium enhancement (LGE) sequence has developed as a tool to evaluate fibrosis in the atrial walls. LA remodelling and fibrosis is theorized to precede the onset of and to increase with the persistence of AF. Pathological studies have demonstrated that other cardiac diseases are associated with LA fibrosis regardless of the presence of AF. Identifying conditions associated with LA LGE in the general population could be important for risk stratification of atrial fibrosis and AF development in patients with such comorbidities. This study aims to identify common clinical conditions associated with greater LA LGE.

## Methods

We retrospectively identified and reviewed the medical records of 181 consecutive subjects imaged with cardiac MRI on a Siemens 1.5T from 2012 to 2013 at our institution. Subjects with age less than 21, prior ablation, or prior sternotomy were excluded. Of the remaining 148 subjects, those were excluded who did not have a 3D LGE sequence or if it was not interpretable. LGE in the LA was described by its presence in 18 locations, resulting in a semi-quantitative score. Measurements of the LA end diastolic volume (LAEDV) and LA end systolic volume were approximated by biplane area-length and used to quantitate LA ejection fraction (EF). Clinical conditions of hypertension, hyperlipidemia, congestive heart failure (CHF), diabetes, obesity, AF, other cardiac disease, and smoking status were identified. Patient medication usage was also determined. Data were analyzed using linear regression, t-tests, and analysis of covariance using JMP v10 (SAS Institute Cary NC).

## Results

**Figure 1:**
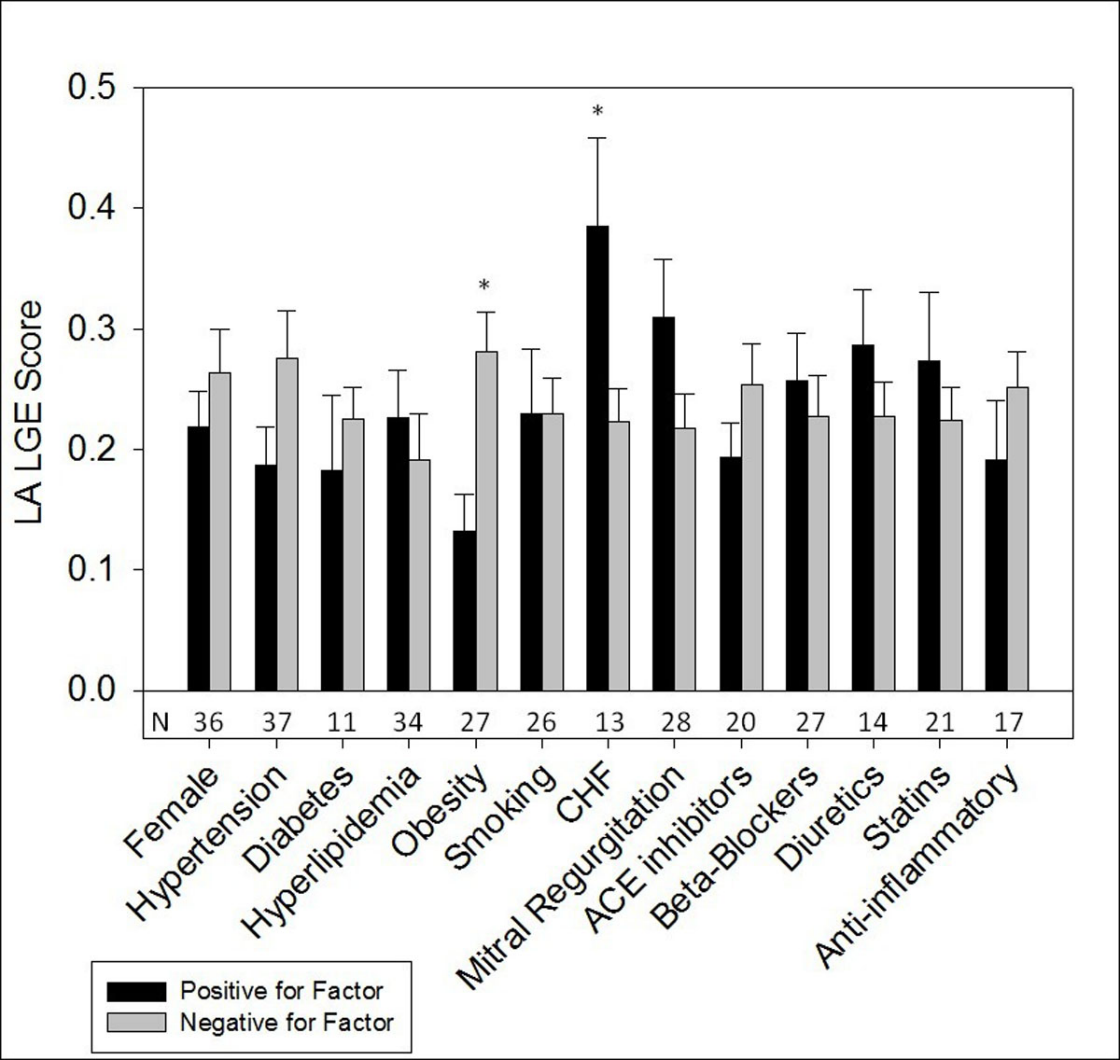
**Clinical factors and association with LA LGE score.** Obese subjects had lower LA LGE score while subjects with congestive heart failure had higher LA LGE score (p<0.05). Number under bar indicates subjects positive for each factor. Values are mean +S.E. Overall N=88.

**Figure 2:**
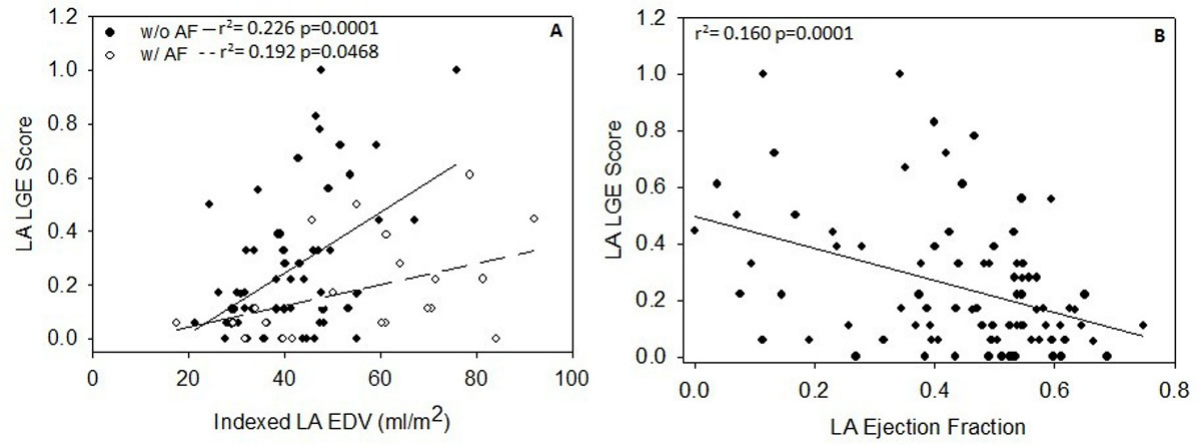
Left atrial end diastolic volume (A) and ejection fraction (B) are mildly associated with left atrial LGE.

Subject (N=88) characteristics consisted of 41% female, mean age 50.8±14.8, BMI 28.8±6.4 (Figure 1). LA LGE was associated with LAEDV index in subjects both with and without AF (r^2^= 0.192, r^2^= 0.226 respectively, p<0.05; Figure 2). Analysis of covariance demonstrated a steeper relationship in patients without AF (p=0.039). Decreased LA EF was associated with increased LA LGE (r^2^=0.16 p<0.001). After accounting for the effects of LAEDV, subjects with CHF had higher LA LGE scores (p=0.039) while obese subjects had lower LA LGE scores (p=0.005). Hypertension, hyperlipidemia, diabetes, age, sex, medication usage, and smoking were not significantly associated with differences in LA LGE.

## Conclusions

Among this subject cohort, the strongest predictor of LA LGE was LA EDV. Additionally, LA EF showed an association with LA LGE. Atrial fibrosis is thought to develop with many cardiac diseases. Accordingly, we found higher LA LGE scores in subjects diagnosed with CHF after accounting for LA volumes. This suggests that the higher LA LGE in CHF subjects was not due to LA volume increases alone. Interestingly, obese patients had lower LA LGE score than non-obese patients. It is unclear whether this is related to a true protective effect of obesity and requires future investigation.

## Funding

This work was partly funded by NHLBI R21 HL 098573.

